# Sequence-Based Explainable Hybrid Song Recommendation

**DOI:** 10.3389/fdata.2021.693494

**Published:** 2021-07-28

**Authors:** Khalil Damak, Olfa Nasraoui, William Scott Sanders

**Affiliations:** ^1^ Knowledge Discovery and Web Mining Lab, Department of Computer Science and Engineering, University of Louisville, Louisville, KY, United States; ^2^ Department of Communication, University of Louisville, Louisville, KY, United States

**Keywords:** song recommendation, hybrid recommender system, recurrent neural networks, explainability, item cold start problem, deep learning, collaborative filtering, transparency and fairness in AI

## Abstract

Despite advances in deep learning methods for song recommendation, most existing methods do not take advantage of the sequential nature of song content. In addition, there is a lack of methods that can explain their predictions using the content of recommended songs and only a few approaches can handle the item cold start problem. In this work, we propose a hybrid deep learning model that uses collaborative filtering (CF) and deep learning sequence models on the Musical Instrument Digital Interface (MIDI) content of songs to provide accurate recommendations, while also being able to generate a relevant, personalized explanation for each recommended song. Compared to state-of-the-art methods, our validation experiments showed that in addition to generating explainable recommendations, our model stood out among the top performers in terms of recommendation accuracy and the ability to handle the item cold start problem. Moreover, validation shows that our personalized explanations capture properties that are in accordance with the user’s preferences.

## 1 Introduction

Among the diverse domains in which automated recommendations play an important role is music. In music, like in other domains, the most accurate recommender systems have been relying on increasingly complex (black-box) machine learning models that cannot explain their output predictions. Hence, one main challenge in designing a recommender system is mitigating the trade-off between recommendation performance (i.e., prediction accuracy) and the ability to explain predictions (i.e., explainability) (Abdollahi and Nasraoui, 2017). State-of-the-Art techniques in music recommendation include Matrix Factorization (MF)-based approaches (Mehta and Rana, 2017) and Deep Learning (DL) architectures (Zhang et al., 2017). MF builds a model that captures the similarities between users and items in a latent space obtained by factorizing the rating matrix into user and item latent factor matrices (Koren et al., 2009). Among all deep learning architectures, deep sequence models (Hochreiter and Schmidhuber, 1997; Cho et al., 2014; Lipton, 2015) are designed to model sequential data. Sequence model-based recommender systems follow three main approaches. The first approach uses sequence models to predict the next interaction given the previous interactions (Hidasi et al., 2015; Hidasi et al., 2016; Tan et al., 2016; Wu et al., 2016; Smirnova and Vasile, 2017) in a session-based fashion. The second approach uses sequence models to model the temporal dependencies, in terms of seasonal evolutions of items and user preferences, to generate recommendations (Wu et al., 2017a; Wu et al., 2017b). Finally, the third approach uses sequence models as a feature representation learning tool on textual data (Bansal et al., 2016). Despite the advances in deep learning for song recommendation and even though the sequential nature of songs makes them naturally amenable to sequence models, no work has used sequence models with the *content* of songs for recommendation. Furthermore, current black-box music recommender systems cannot explain their recommendations based on content. On music streaming platforms, new users and songs are constantly added, and since these additions have few, if any, ratings, they cannot be handled by classical CF algorithms. This problem, known as the *cold start problem*, thus adds another challenge to collaborative filtering (CF) recommender systems in addition to the demands of recommendation accuracy and explainability (Abdollahi, 2017).

### 1.1 Contributions

In this work, we take advantage of the sequential nature of songs’ content, the prediction power of MF, and the superior capabilities of DL sequence models to present the following contributions:

• We propose a method to transform the Musical Instrument Digital Interface (MIDI) format of songs into multidimensional time series to be the input for sequence models and, hence, capture rich information about the song;
• We integrate content-based filtering using DL sequence models with CF to build a hybrid model that provides accurate predictions compared to state-of-the-art CF recommender systems, while also providing personalized explanations and handling the item cold start problem
• We propose a new type of content-based explanation that consists of presenting a short personalized MIDI segment from the song that characterizes the portion that the user is predicted to like the most;
• We present two evaluation methodologies of the personalized music explanation segments based, respectively, on the concordance of musical sound and the preferred user tags. Given the absence of any prior technique for song explanation based on segments, our evaluation approach attempts to evaluate why a song’s segment serves as an explanation for a given user; and
• We perform an online user study that demonstrates the validity of personalized segment explanations and their ability to improve user satisfaction, effectiveness, and transparency.

## 2 Related Work

### 2.1 Sequence Models in Recommendation

Various recommender systems rely on sequence models (Wang et al., 2019a; Wang et al., 2019b). However, not all of them use them for recommendation with user preferences. In fact, some are session-based CF models that predict the next interaction (Hidasi et al., 2015; Tan et al., 2016; Wu et al., 2016; Hidasi and Karatzoglou, 2018; Yuan et al., 2020), or basket of interactions (Yu et al., 2016; Wang Z. et al., 2018; Wang et al., 2020a; Wang et al., 2020b), in a sequence of interactions regardless of the user’s personal preferences. Similarly, other approaches relied on self-attention networks (Vaswani et al., 2017) to predict the next item recommendation given a sequence of consecutive interactions (Kang and McAuley, 2018; Sun et al., 2019; Li et al., 2020; Tan et al., 2021). Other methods integrated content into session-based recommendation (Hidasi et al., 2016; Smirnova and Vasile, 2017) and proved that side information enhances the recommendation quality (Zhang et al., 2017). Other sequence-model-based recommender systems take into consideration the user’s identification (Wu et al., 2017a; Wu et al., 2017b). These engines model temporal dependencies for both users and movies (Wu et al., 2017a; Wu et al., 2017b) and generate reviews (Wu et al., 2017a). The main objective of the aforementioned models is to predict ratings of users to items using seasonal evolutions of items and user preferences in addition to user and item latent vectors. Alternative models aimed to generate review tips (Li et al., 2017), predict the returning time of users, and predict items (Jing and Smola, 2017) or produce next item recommendations for a user by using a novel Gated Recurrent Unit (Cho et al., 2014) (GRU) structure (Donkers et al., 2017). Finally, some recommender systems use sequence models as a feature representation learning tool for text recommendation (Zhang et al., 2017). For instance, (Bansal et al., 2016), created a latent representation of items and used it as input to a CF model with a user embedding to predict ratings.

Our proposed approach differs from the aforementioned recommender systems in the goal towards which the sequence model is used. In fact, as opposed to the other approaches, we use a sequence model to encode the sequential evolution of the song content, and leverage this kind of information later in the rating prediction process.

### 2.2 Hybrid Song Recommender Systems

In contrast to all the aforementioned efforts, song recommendation has attracted the attention of only a few hybrid models, that differ significantly from one another in terms of the input data and the features created. In fact, music items can be represented by features derived from audio signals, social tags or web content (Vall and Widmer, 2018). Among the most noticeable hybrid song recommender systems, (Wang and Wang, 2014) learns latent factors of users and items using matrix factorization and then sums their product with the product obtained from the constructed user and song features. Meanwhile, (Benzi et al., 2016) combines non-negative MF and graph regularization to predict the inclusion of a song in a playlist. Another approach (Oramas et al., 2017) learns artist embeddings from biographies and track embeddings from audio spectrograms, and then aggregates and multiplies them by user latent factors obtained by weighted MF to predict ratings. Van den Oord et al. (2013) trains a Convolutional Neural Network (LeCun et al., 1999) on spectrograms of song samples to predict latent features for songs with no ratings. Finally, Andjelkovic et al. (2018) positions the users in a mood space, given their favorite artists, and recommends new artists using similarity measures.

### 2.3 Explainability in Recommendation

According to Arrieta et al. (2020), explainability can either come from transparent models or post-hoc techniques that try to explain predictions after they are generated. Explaining recommendations using transparent models can vary from using simple user or item-based (Sarwar et al., 2001) CF approaches that rely on rating matrix similarities, to building white-box models (Zhang and Chen, 2018). The methods that are most related to our work rely either on MF or deep learning. Among the MF-based white-box models, we find (Abdollahi and Nasraoui, 2017), which optimizes a measure of explainability with the recommendation accuracy yielding explainable recommendations with user or item-based neighbor style explanations. Coba et al. (2019) and Wang S. et al. (2018) extended the idea by, respectively, trying to improve the novelty of the recommendations and modifying the calculation of the explainability matrix by integrating the neighbors’ weights. Other works (Zhang et al., 2014; Zhang, 2015) used sentiment analysis on user review data along with MF-learned latent features to generate explainable recommendations. The explanations, in this case, are in the form of either user or item features (Zhang et al., 2014), textual sentences (Zhang et al., 2014), or word clusters (Zhang, 2015). On the other hand, among deep learning-based explainable models, we find (Chen et al., 2018) which uses memory-based structures, such as sequence models, to introduce users’ historical records into a MF-based model. The explanations in this case are generated using an attention mechanism (Luong et al., 2015) in sequence models which provide insight on how the user’s historical records affect their current and future decisions (Chen et al., 2018). For instance, (Seo et al., 2017), models user preferences and item properties using attention-based (Luong et al., 2015) Convolutional Neural Networks (CNNs) (Lecun et al., 1998) for review rating prediction. The explanation, in this case, is an importance heatmap of each word in the review. On the other hand, (Li et al., 2017), proposes a multimodal attention network that explains fashion recommendations using image regions and their correspondences with the user’s review. Because there is usually a tradeoff between explainability and recommendation accuracy, some research has focused on post-hoc explanainability of powerful black-box models. Such work includes (Rastegarpanah et al., 2017) which explains MF-based recommender systems using influence functions to determine the effect of each user rating on the recommendation. Cheng et al. (2019) also uses an influence-based approach to measure the impact of user-item interactions on a prediction and provides neighborhood-style explanations. Finally, Wang X. et al. (2018) proposes a model-agnostic reinforcement learning framework that was demonstrated with sentence-level explanations.

We propose a model-specific post-hoc explainable recommender system (Arrieta et al., 2020) that, aside from reaching competitive recommendation performance compared to state-of-the-art methods, succeeds in explaining a song recommendation using a personalized 10-second instrumental segment from the recommendation.

## 3 Methods

### 3.1 Data Preparation for MIDI Content and Ratings

To build a dataset that includes both user to item interactions and song content data, we used two datasets from the Million Song Dataset (MSD) (Bertin-Mahieux et al., 2011). The Echo Nest Taste Profile Subset (Bertin-Mahieux et al., 2011) includes 48, 373, 586 play counts of 1, 019, 318 users to 384, 546 songs collected from The Echo Nest’s undisclosed partners. The Lakh MIDI Dataset, on the other hand, includes 45, 129 unique MIDI files matched to the MSD songs (Raffel, 2016a; Raffel, 2016b). We combined both datasets by taking the intersection in terms of songs. Then, we followed the same methodology used in He et al. (2017) to reduce the sparsity of the data, and filtered out users that interacted with fewer than 20 unique songs. Consequently, we ended up with a dataset consisting of 32, 180 users, 6, 442 songs with available MIDI files, and 941, 044 play counts.

**FIGURE 1:**
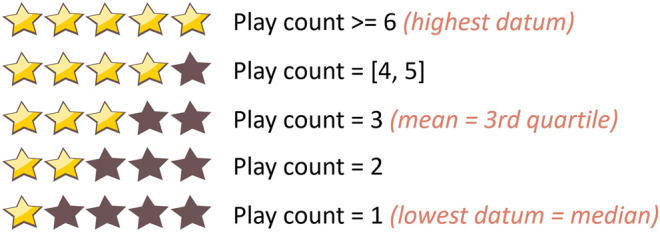
Play count normalization into 5-star ratings.

**FIGURE 2:**
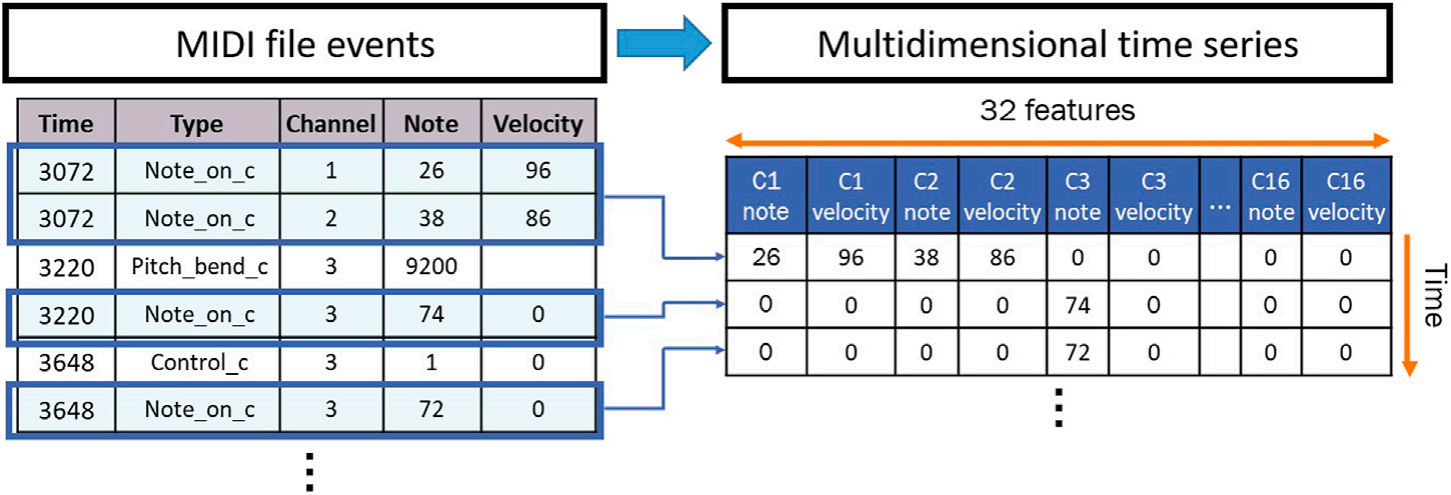
MIDI events to multidimensional time series transformation.

We pre-processed our dataset by first mapping the play counts to ratings to remove outliers. To do so, we used the statistics of the play counts to map them to ratings as shown in Figure 1. Next, we created the inputs to train sequence models by transforming each MIDI file into a multidimensional time series. MIDI files are polyphonic digital instrumental audios that are used to create music. They are composed of event messages that are consecutive in time^1^. Each message includes a type (such as a note), notation (the note played), time (the time it is played), and velocity (how rapidly and forcefully it is played). These events are distributed over 16 channels of information, which are independent paths over which messages travel^1^. Each channel can be programmed to play one instrument. We first used “MIDICSV”^2^ to translate the MIDI files into sheets of the event messages. We only considered the “Note on C” events to focus our interest on the sequences of notes played throughout time. In fact, the “Note on C” event represents the event of a note being played. It includes features such as the note being played, its velocity, the channel of information, and the time stamp during which it is being played. Thus, we extracted the notes that are played within the 16 channels with their velocities. As a result, each transformed multidimensional time series consists of a certain number of rows representing the number of “Note on C” events and 32 features representing the notes and velocities played within the 16 channels. The transformation process is summarized in Figure 2.

We then normalized the number of time steps to the median number of time steps of the songs in our dataset (2,600) to be able to train models with mini-batches (Li et al., 2014). To avoid duplicates of the same song in the input and ensure memory efficiency, we created a song lookup matrix by flattening each multidimensional time series into one row in this matrix.

### 3.2 Sequence-Based Explainable Recommender System

**FIGURE 3:**
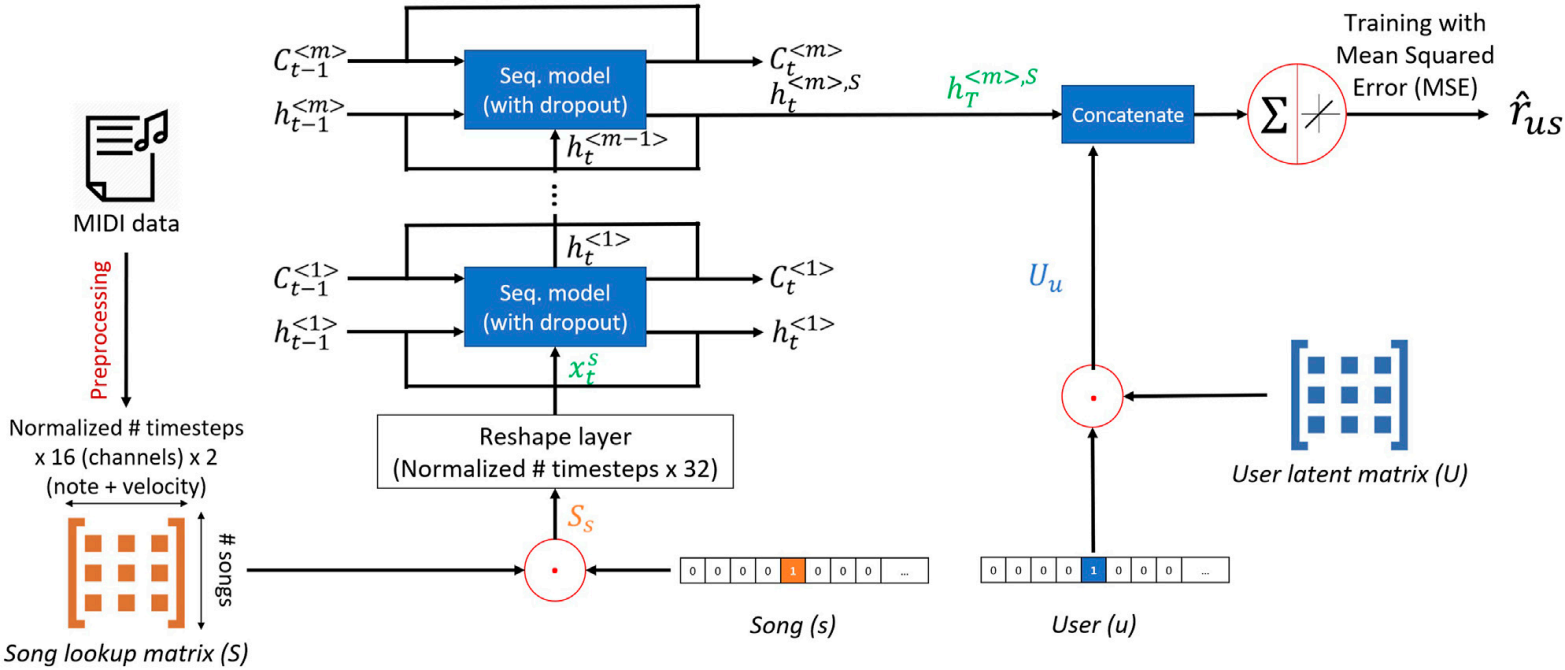
Structure of SeER: For every training tuple (user, song, rating), the model extracts the corresponding user latent vector and flat song array from the user latent matrix and the song lookup matrix, respectively. The song array is reshaped to its original 2-dimensional format and input to a sequence model. The resulting song’s hidden state vector is concatenated to the user’s latent vector and the predicted rating is a weighted combination of the resulting vector.

We designed a model, that we call “SeER”: a sequence-based explainable hybrid song recommender system (Figure 3), which takes as input the song lookup matrix and a user embedding matrix. The user embedding matrix consists of learnable weights that are randomly initialized, and are updated during the model training to represent hidden characteristics of the users. For each user, song, and rating triplet $\left(u,s,r_{us}\right)$ in the training data *R*, we extract the corresponding latent factor vector $U_{u}$ of the user and the flattened song array $S_{s}$. The latter process is illustrated in Figure 3 with multiplications of the user embedding and song lookup matrices with one hot vectors of *u* and *s* respectively. The song array is next reshaped into its two-dimensional original shape (2,600 time steps by 32 features). The resulting array $x^{s}$ is input to a sequence model and, finally, the last layer ($m^{th}$ layer) at the last time step (T=2,600), produces the hidden state $h_{T}^{\langle m\rangle ,s}$, which is concatenated to the user’s latent vector $U_{u}$ and then used as input to a neuron with a linear activation to predict a rating of the user to the song such that ${\hat{r}}_{us}=a\left[U_{u},h_{T}^{\langle m\rangle ,s}\right]$. Where (.,.) represents a concatenation and *a* is a weight vector. The intuition is that the weights $\left[a_{k}\;\left|\;k\epsilon 1..\right|\left(U_{u},h_{T}^{\langle m\rangle ,s}\right)|\right]$ would regulate the flow of information coming from the user’s latent factor and the hidden state, which is a representation of the song’s content, to predict the rating. We chose the size of the hidden state to be the same as the number of user latent features to constrain the model to represent the user and the song in the same latent space size. The model is trained using the Mean Squared Error (MSE) (Lehmann and Casella, 1998), with the loss between the actual rating $r_{us}$ and the predicted rating ${\hat{r}}_{us}$, given by:

$$J_{SeER}=\frac{1}{\left|R\right|}\underset{\left(u,s,r_{us}\right)\epsilon \text{R}}{\sum }{\left({\hat{r}}_{us}-r_{us}\right)}^{2}\;=\frac{1}{\left|R\right|}\underset{\left(u,s,r_{us}\right)\epsilon \text{R}}{\sum }{\left[a\left[U_{u},h_{T}^{\langle m\rangle ,s}\right]-r_{us}\right]}^{2}$$ (1)

Note that in Figure 3, the cell states $C_{t}^{<m>}$ can be ignored when using Recurrent Neural Networks (Lipton, 2015) (RNNs) or Gated Recurrent Units (Cho et al., 2014) (GRUs).

### 3.3 Segment Forward Propagation Explainability

After generating a song recommendation *s* to a user *u*, we explain it by presenting a 10-second MIDI segment $x_{u}^{s,exp}$ of the song that strives to justify the recommendation using the most important portion of the recommended song for the user. To do so, we sample 10-s segments from the recommended song array $x^{s}$ using a sliding window of 1 s. Then, we input the segments along with the user’s latent vector $U_{u}$ to the trained model to predict a rating for each segment. Finally, the segment with the highest predicted rating is selected as the explanation for the song recommendation. The insight is that the segment with the highest predicted rating is the segment that is predicted by the model to best match the preferences of the user. Thus, it could be considered as the segment that had the most influence on the rating prediction of the song for the user. That is why we rely on it to explain the recommendation. The explanation process is summarized in Algorithm 1. To illustrate the SeER recommendation and explainability processes, we provide a link to a video^3^ demo that demonstrates the top 10 explained recommendations for user 1,000 in our dataset.

Note that the approach of learning on entire objects and then explaining using sub-objects is intuitive and commonly used in classifying objects that can be decomposed (e.g., using regions or pixels for images or words for text). We relied on the same intuition to design our Segment Forward Propagation Explainability mechanism which extends the mechanism to the music content. Also note that the MIDI format of the explanations is intended to match the type of content that the model used to generate the recommendations. Thus, when the user listens to the MIDI-based explanation, they would understand that the song recommendation was based on the MIDI (melodial) segment presented regardless of any other type of content such as the lyrics. Finally, since this is a new approach to explain song recommendations, we could not rely on any known standards for selecting the optimal segment length. Our choice of 10 s for the length of the explanation is largely justified by the fact that 10 s song previews are common on music platforms. Moreover, 10 s seemed long enough to form a consistent explanation but still short enough to constitute a small portion of a song. We leave studying the effectiveness of various sequence lengths to future work.

ALGORITHM 1 Segment Forward Propagation Explainability

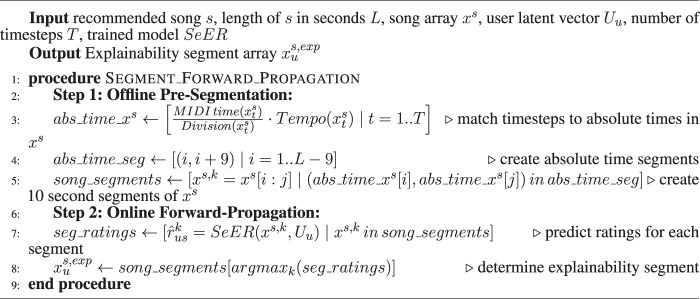


## 4 Offline Experimental Evaluation

In this section, we assess the proposed model’s recommendation accuracy and ability to handle the item cold start problem by comparing it to state-of-the-art baselines. Then we validate the explanation segments with offline experiments.

### 4.1 Experimental Setting

We used the same 80/20% train/test split for all the experiments. We report the best results within 20 epochs in terms of recommendation ranking using Mean Average Precision at cutoff K (MAP@K) and Normalized Discounted Cumulative Gain at cutoff K (NDCG@K). We also compared the models in terms of rating prediction using the Root Mean Squared Error (RMSE). The code, data, and trained models are available for reproducibility^4^.

### 4.2 Hyperparameter Tuning

We fixed the number of sequence model layers to one and the batch size to 500 due to our limited memory budget. Also, we relied on the Adaptive Moment Estimation (Adam) (Kingma and Ba, 2014) optimizer. Finally, we tuned the number of latent features from 50 to 200 with increments of 50, the sequence model type by trying RNN, GRU, and Long Short-Term Memory (Hochreiter and Schmidhuber, 1997) (LSTM) and, finally, the normalized sequence lengths within the set {2,600, 1,000, 500, 300, 100}. We relied on a greedy approach, that consists of varying the hyperparameters one by one independently from each other. Note that tuning the sequence length aims to avoid overfitting and vanishing gradient issues due to long-term dependencies. We reached the best performance with 150 latent features, LSTM as the sequence model and a sequence length of 500.

### 4.3 Research Questions

To evaluate the prediction ability of our model, we match it to state-of-the-art baseline recommender systems regardless of their types and nature of input data. This leads us to formulate our first research question:

**RQ1:** How does our model compare to baseline recommender systems?

In addition, we run experiments to demonstrate how well our model overcomes the item cold start problem compared to pure CF models in the second research question:

**RQ2:** How well does our model solve the item cold start problem?

Finally, we assess whether our explanations share similar characteristics based on pure MIDI content. The logic behind this is that the shared characteristics may be interpreted as user preferences that could be captured in the explanations. This is translated in the following question:

**RQ3:** Do the personalized explanations share similar characteristics?

### 4.4 RQ1: How Does Our Model Compare to State-of-the-Art and Baseline Recommender Systems?

We compare against the following recommender system models, which include competitive state-of-the-art models (i.e., the top 2 performing models on the MSD data^5^) as well as simpler baselines:

1) **Matrix Factorization (** Mehta and Rana, 2017 **):** This is one of the most common CF techniques. We optimize its loss with Stochastic Gradient Descent, and tuned the number of latent factors from 50 to 200 with an increment of 50.
2) **NeuMF (** He et al., 2017 **):** This is a state-of-the-art CF technique that combines Generalized Matrix Factorization (He et al., 2017) and Multi-Layer Perceptron (LeCun et al., 1988). We replaced its output layer with a dot product and used MSE as a loss function because we are using ratings. We followed the same tuning process that was employed in He et al. (2017).
3) **RecVAE (** Shenbin et al., 2020 **):** This is a state-of-the-art variational autoencoder-based implicit feedback recommender system. It is the second to best model in ranking performance on the MSD data according to^5^. We fixed the hyperparameters to the values recommended for the MSD in Shenbin et al. (2020), and tuned the latent dimension from 50 to 200 with increments of 50.
4) **EASE (** Steck, 2019 **):** This is a linear model based on shallow autoencoders. It is the best model in terms of ranking performance on the MSD dataset according to^5^. It is an implicit feedback model. We tuned the regularization parameter *λ* within the set of values {0.5, 1, 100, 200, 500, 1,000}.
5) **ItemPop (** Rendle et al., 2009 **):** This is the most popular item recommendation model, a simple baseline to benchmark the performance.

For each of the implicit feedback models, we converted the ratings into either interactions or normalized ratings in the same way described in their respective papers and compared the results in terms of ranking performance. We present the average results over five replications obtained with each model in Table 1. We also applied a Tukey test (Haynes, 2013) for pairwise comparison for each metric and report the group-ranked results, categorized into groups ordered from A to D, from best to worst performance, that we list next to the average performances. For instance, models in group A are not significantly different from each other, but they are significantly different from models in group B; and so forth. SeER yielded the best MAP@5 and NDCG@10 scores of 0.4145 and 0.9867 respectively, and was second to best in terms of MAP@10 and NDCG@5 following EASE which is known as the best performing model so far on the MSD dataset^5^. It is worth noting that in the latter two metrics, even though SeER was second to best, the difference with EASE was not significant, as both models were in group A and were significantly better than all the other models. Furthermore, SeER presented significantly better rating prediction performance, in terms of RMSE, than all the other models (i.e., SeER was in group A while the other models were in groups B and C). Note that models belonging to group (A/B) can, statistically, be considered in either group. It is important to mention that in addition to its competitive recommendation accuracy, SeER can handle the cold start problem, as we show next, and has the unique ability to explain its recommendations. Hence, our approach is able to mitigate the cold start problem and provide explanations while still achieving state-of-the-art performance.

**TABLE 1:** Comparison of SeER with baseline models: Average performance over five replications. **Best scores are in bold** and second to best scores are underlined. First three models are implicit feedback models, and last three are explicit feedback models. Tukey test groups are between parenthesis, ordered from A (best) to D (worst). Based on the group based ranking, SeER ranks in the top performance group (A).

Model	MAP@5	MAP@10	NDCG@5	NDCG@10	RMSE
RecVAE	0.3622 (C)	0.4082 (C)	0.8328 (C)	0.9841 (C)	—
EASE	0.4144 (A/B)	**0.4554(A)**	**0.8535(A)**	0.9863 (A/B)	—
ItemPop	0.0978 (D)	0.1450 (D)	0.0566 (D)	0.0752 (D)	—
MF	0.3598 (C)	0.4057 (C)	0.8338 (C)	0.9842 (C)	2.4977 (C)
NeuMF	0.4109 (B)	0.4515 (B)	0.8482 (B)	0.9855 (B)	1.2765 (B)
SeER	**0.4145(A)**	0.4550 **(A)**	0.8528 **(A)**	**0.9867(A)**	**1.2433(A)**

### 4.5 RQ2: How Well Does Our Model Solve the Item Cold Start Problem?

Even with no ratings, *unseen* songs can have their MIDI content propagated through the sequence model, thus allowing SeER to handle the item cold start problem. We validate the robustness to the item cold start problem by splitting the dataset into training and test sets in terms of songs. Specifically, we randomly hold out ratings related to 5% (46,069 ratings), 10% (92,347 ratings), 15% (143,535 ratings), and 20% (191,159 ratings) of the songs from the training set and use the held out songs as a test set. We made sure to include ratings from all the users in the training set to avoid user cold start issues. We assess the prediction capacity of SeER compared to only the baseline *explicit* feedback models because we cannot be guaranteed to have enough items for all the users in the test data to compute ranking metrics. Additionally, we compare to a Multi-Layer Perceptron (MLP) architecture with the same hyperparameter configuration as in the MLP part of NeuMF. Table 2 compares the average RMSE over five replications for varying item cold start levels for all the explicit feedback models. To predict a rating of an unseen song, the four models rely on the learned user’s latent vectors. However, in contrast to SeER, MF, MLP, and NeuMF combine the user’s latent vector with the un-updated (thus the randomly intialized) song’s latent vector to generate the output. SeER has the unique ability to also employ the song’s content as input to the learned sequence model and then combines the user’s latent vector with the resulting item’s hidden state to predict the rating. The results in Table 2 demonstrate how SeER significantly outperforms the other baselines, namely MF, MLP, and NeuMF, for all the item cold start settings. This means that our proposed approach is more robust in dealing with unseen items, and demonstrates its ability to mitigate the item cold start problem by relying on the content of songs.

**TABLE 2:** Average RMSE over five replications. We compare SeER to only *explicit* feedback models (MF, NeuMF, and MLP) because we cannot guarantee to have enough items for all the users in the test data to compute ranking metrics. SeER achieves a lower RMSE compared to the other approaches, for increasing item cold start levels, which means that it is more robust in dealing with unseen items. All differences are significant (Tukey test p−values<0.05).

	% Item cold start				
	0% (no cold start)	5%	10%	15%	20%
MF	2.4977	2.5696	2.5344	2.5100	2.5487
NeuMF	1.2765	1.3273	1.3166	1.2889	1.3237
MLP	1.2750	1.3123	1.3046	1.2750	1.3017
SeER	**1.2433**	**1.3055**	**1.2914**	**1.2652**	**1.2940**

### 4.6 RQ3: Do the Personalized Explanations Share Similar Characteristics That Capture User-Preferences?

In order to validate the 10-s segment explanations, we tried to determine, for every user, whether their personalized explanations share some common characteristics. This is because explanations that share common properties are likely to have been generated based on user preferences that have been learned by the model. Hence, they may represent relevant sections of the recommended songs instead of being just artifacts. To study the latter property, we propose two approaches based on analysis of the concordance between song content and tags.

#### 4.6.1 MIDI Content-Based Validation

We computed distance measures between the explanations’ MIDI content to prove that they share similar characteristics. We randomly selected 100 test users, for whom we generated the top five recommendations and their explanations. We then computed the average Dynamic Time Warping (DTW) (Salvador and Chan, 2007) distance between the explanations (DTWe), which can compare multidimensional time series that do not necessarily have the same size. To compare two lists of multidimensional time series, we compute the DTW distance matrix between them and take the average of all the values in the matrix. In the case of DTW distances between explanations, both lists are similar and include the song arrays of the explanation segments for the top-5 recommended songs. As a comparison baseline, we selected a random 10-s segment from every recommended song and computed the average DTW distance between these five segments (DTWr) for every user. Note that we compute the average DTW distances between 10-s segments instead of between the entire recommended songs to avoid any bias caused by the different song lengths. Finally, we considered the problem as a Randomized Complete Block Design (RCBD) (Olsson, 1978) and applied a Tukey test (Haynes, 2013) for pairwise comparison. **The null hypothesis** is that when averaged over all the users, the average DTW distances between the explanations (DTWe) and average DTW distances between the random segments (DTWr) are similar. For simplicity, we will call these two quantities “Avg. DTW between explanations” (or $\bar{DTWe}$) and “Avg. DTW between random segments” (or $\bar{DTWr}$). We show these average values with the 95% Confidence Intervals (CIs) of the difference ($\bar{DTWe}-\bar{DTWr}$) for SeER and the corresponding statistical test results in Table 3. We notice that $\bar{DTWe}$ is significantly smaller than $\bar{DTWr}$ (*p*-value <0.05 and 0 is not in the Confidence Interval). This means that for each user, we can assert with 95% confidence that the explanations are significantly close to each other compared to the random segments. Thus, we can assert that our generated 10-s segment explanations share some common characteristics which are likely to represent the learned preferences of the user.

**TABLE 3:** Significance testing with 95% confidence of the difference between Avg. DTW between explanation and Avg. DTW between random segments: The explanations are significantly close to each other compared to the random segments. This means that the explanations capture and share some common characteristics that are likely to represent the learned user’s preferences.

Avg. DTW between explanations ($\bar{DTWe}$)	Avg. DTW between random segments($\bar{DTWr}$)	95% CI of difference($\bar{DTWe}-\bar{DTWr}$)	Adjusted *p*-value
**7,949.2**	8,467	(25, 1,010)	**0.04**

#### 4.6.2 Tag-Based Validation

In addition to pure music content, tags can capture an item’s properties in terms that are familiar to humans. In the case of songs, they can include genres, the era, the name of the artist, or subjective emotional descriptions. We used the tags from the “Last.fm” dataset (Bertin-Mahieux et al., 2011) provided with the MSD. These tags are available for almost every song in the MSD and amount to 522,366 tags (Bertin-Mahieux et al., 2011). In our dataset, we selected the songs that intersect with the “Last.fm” dataset and filtered the tags that occur in at least 100 songs in order to remove noisy tags. We obtained 4,659 songs with 227 tags. From the users that interacted with these songs, we filtered the ones that have at least 10 liked songs with the assumption that a rating strictly higher than three means that the user likes the song. Next, we randomly selected 100 users as our test sample. For every user, we determined the top 1, 2, and 3 preferred tags, based on the tags of their liked songs, and generated the top five recommendations with explanations using SeER.

Our objective is to determine how much the personalized recommendations and explanations match the preferred tags of every user. Thus, we needed to determine the tags of both the recommendations and the explanations, which are not necessarily in the tag dataset. To cope with this issue, we trained a multi-label classification model on our tagged dataset to predict the tags of the recommendations and explanations. The classifier is a sequence model layer with 20% dropout, followed by Multi-Layer Perceptron (MLP**)** (Popescu et al., 2009) layers with ReLU activations and an output layer with 227 nodes, corresponding to the 227 classes (i.e., tags), each with a Sigmoid activation function. The model is trained to optimize the Binary Cross-entropy loss to predict the probability of each tag individually in every node (Lapin et al., 2017).

To tune the tag classification model’s hyperparameters, we started with an LSTM layer followed by the output layer. We tuned the size of the hidden state from 100 to 500 with an increment of 100. Then, we tuned the number of MLP hidden layers from 1 to 5. We chose the number of nodes in the hidden layers to be the optimal size of the hidden state, which is 300. Finally, we tuned the sequence model type of the first layer by additionally trying RNN and GRU. The best model has one LSTM layer with a hidden state size of 300 followed by four MLP layers of the same size and, finally, the output layer. We reached a performance of 93.4% accuracy; and respectively, 51.8, 61.9, and 67.7% top-1, top-2 and top-3 categorical accuracy with 5-fold cross validation. We used top-k categorical accuracy (Lapin et al., 2017) because we are interested in correctly predicting the existing tags in a sparse target space. We used our trained tag classifier to predict the tags of all the recommendations and explanation segments for all the users. Then, we calculated the Average Percentage Match of the recommendations and explanations with the top 1, 2, and 3 user preferred tags.

We define the Percentage Match of a list of songs *S* with the top *k* preferred tags $T_{k}\left(u\right)$ of a user uϵU as the percentage of songs from *S* including at least one of the top *k* preferred tags $T_{k}\left(u\right)$, as follows:

$$\text{\%}\;Match\left[S,T_{k}\left(u\right)\right]=\frac{100}{\left|S\right|}\left|\left(s\epsilon S|Tags\left(s\right)\cap T_{k}\left(u\right)\neq \emptyset \right)\right|$$ (2)

Tags(s) is the set of tags of the song *s*. In our case, the set of tags of a recommendation or an explanation is predicted using the multi-label classification model. The Average Percentage Match over all the test users is computed using:

$$Avg\;\text{\%}\;Match\left(S,U,k\right)=\frac{100}{\left|U\right|}\overset{\left|U\right|}{\underset{u=1}{\sum }}\text{\%}\;Match\left(S\left(u\right),T_{k}\left(u\right)\right)$$ (3)

S(u) is either the set of recommendations or explanations for user *u*. We varied *k*, considered every problem as a Randomized Complete Block Design (RCBD) (Olsson, 1978), and applied Tukey tests (Haynes, 2013) for pairwise comparison. The null hypothesis for every test is whether the average percentage match of the recommendations and of the explanations with the top *k* liked songs (Avg%Match (rec., U, k) and Avg%Match (exp., U, k), respectively) are equal. We show the two average percentage match values with the corresponding 90% CIs of the differences (Avg%Match (rec., U, k) - Avg%Match (exp., U, k)) and adjusted *p*-values of the Tukey tests in Table 4. We notice that for all k, the explanations match the preferred tags of the users more than the recommendations. The difference is significant for k = 1, 2, and 3 (CI of the difference does not include 0 and *p*-value <0.1). This means that the explanations share similar properties which agree with the preferred tags of the users even more than the overall recommendations. For instance, assuming that the tags represent the genres, if the user’s preferred genre is, for instance, “Rock,” and a “Pop” song gets recommended, the explanation of that song is likely to be a “Rock” segment of the song, which means that the explanations are personalized. We show an example of a user from our test sample in Table 5.

**TABLE 4:** Significance testing with 90% confidence of the difference between the Avg % Match of recommendations and explanations with user top k preferred tags. The results show that explanations can tell more about the recommendation since they capture a user’s expressed tag preferences.

k	Avg%Match (rec., U, k)	Avg%Match (exp., U, k)	90% CI of the difference	Adjusted *p*-value
1	83.43%	**85.85%**	(−0.0482, −0.0003)	**0.096**
2	94.14%	**94.94%**	(−0.01469, −0.00148)	**0.045**
3	96.16%	**96.96%**	(−0.01469, −0.00148)	**0.045**

**TABLE 5:** Example of a Test User (#26647) where the explanations match the favorite tags more than the recommendations: The first recommended song is a “pop” song (in bold). However, the explainability segment is both “pop” and “rock” which matches the favorite tags of the user better than the recommendation itself (value in bold), thus validating this instance.

Recommendation	Recommendation tags	Explanation tags
1	**Pop**	Pop, rock
2	Pop, rock	Pop, rock
3	Pop, rock	Pop, rock
4	Pop, rock	Pop, rock
5	Pop, rock	Pop, rock
User’s top 3 tags (sorted)	**Rock**, pop, favorites		
**K**	**1**	**2**	**3**
$\text{\%}\;Match\left[rec.,T_{k}\left(u\right)\right]$	**80%**	100%	100%
$\text{\%}\;Match\left[exp.,T_{k}\left(u\right)\right]$	100%	100%	100%

## 5 User Study Evaluation

We performed a real-life user study^6^ that aims to evaluate the validity of our explainability process. We were granted approval from the Institutional Review Board (IRB) before conducting our user study.

### 5.1 Hypotheses and Research Questions

Our hypothesis is that an explanation to a relevant recommendation using our model will lead to better **satisfaction**, **effectiveness**, and **transparency** than a random 10-s segment explanation. First, “satisfaction” measures the contentment of the user with explanations accompanying a set of relevant recommendations based on their ratings. Hence, **RQ5** Does the type of explanation (i.e., personalized vs random) impact user satisfaction with the model?

Moreover, we assess “effectiveness,” which is the ability of the explanation to help the user make good decisions (Abdollahi, 2017). Finally, we evaluate “transparency” which is the comprehensibility of how the model works and its ability to justify the recommendations (Tintarev and Masthoff, 2007). This suggests the following questions:

**RQ6:** Does our type of explanation (i.e., instrumental segment) impact perceived effectiveness of the model?

**RQ7:** Does our type of explanation increase perceived transparency of the model?

### 5.2 Experimental Procedure

The user is presented a list of 100 songs randomly selected from our dataset and is asked to rate at least 10 of them. They are also provided a link to every song so that they can listen to any songs with which they are unfamiliar. Based on the ratings, three recommendations, each with two different explanations, are generated and presented to the user. The first explanation is generated using the Segment Forward Propagation Explainability process while the second explanation is a baseline random 10-second segment of the song. Of course, the subject does not know the difference between the two explanations, they are presented as “EXPLANATION 1” and “EXPLANATION 2” respectively. Each recommendation is accompanied with a related Likert Scale questionnaire. The questions are presented in Table 6 as Questions 1 and 2. They aim to assess the user satisfaction with the explanation compared to the random segment, and thus, answer **RQ5**. Next, a questionnaire with general questions is presented to the user. This aims to assess the effectiveness and transparency criteria defined in the previous subsection in addition to collecting demographic data about the users to describe our sample. The latter questionnaire is presented in Table 6 as Questions 3 to 9. Questions 3 and 4 respectively assess the effectiveness and transparency. Finally, questions 5 to 9 collect demographic data about the users. Note that in the following subsections, the results and statistics might not always match the sample size because users have the choice of not answering a question or not submitting a form.

**TABLE 6:** Survey questions.

Questions related to recommendations	
Question 1	The song segment “EXPLANATION 1” explains why someone would like the song
Question 2	The song segment “EXPLANATION 2” explains why someone would like the song
**General questions**	
Question 3	I will listen to the song based on a 10-s sample that I like
Question 4	The 10-s sample explanation helps me understand how the recommender system works
Question 5	What is your age?
Question 6	What is your sex?
Question 7	What is your major?
Question 8	How familiar are you with automated recommender systems?
Question 9	I cannot spend a day without listening to music

### 5.3 Subject Sample

Participants (N = 30) were recruited through fliers or emails across a large, urban public university. Participant’s age (Mean = 31) ranged from 18 to 54 and there were 12 male and 15 female participants. The majority of participants were Computer Science majors (78%) followed by mathematics (7%) and education majors (15%) respectively. 74% of the volunteers somewhat or strongly agree that they cannot spend a day without listening to music. Moreover, most of the participants (74%) are familiar with recommender systems.

Our choice of the sample size was based on a prior prospective study. Our goal was to determine the minimum sample size necessary to detect a minimum difference between the average measures of satisfaction of the two types of explanation that corresponds to 0.5, with a power of 95%, and assuming a standard deviation of 0.7. In fact, we considered the Likert scale levels as values from 1 to 5 for our statistical tests, where 1 represents “Strongly disagree” and 5 represents “Strongly agree.” The prospective test suggested a minimum sample size of 28, that we rounded up to 30 participants.

### 5.4 Analysis of Results

We evaluate the explainability in terms of satisfaction (Questions 1 and 2), effectiveness (Question 3) and transparency (Question 4).

#### 5.4.1 RQ5: Does the Type of Explanation (i.e., Personalized vs Random) Impact the User Satisfaction?

**FIGURE 4:**
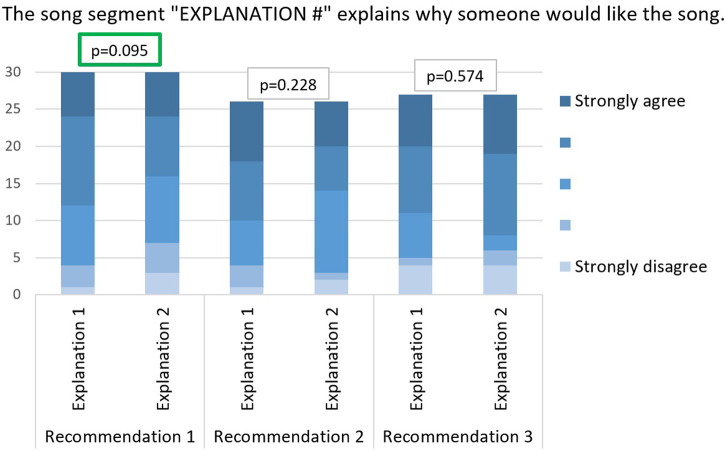
User satisfaction with explainability: Comparison between answers to questions 1 and 2 with paired *t*-test *p*-values.

We compared our explanations (“EXPLANATION 1”) to random 10-s segments (“EXPLANATION 2”) in all three recommendations for every user. The comparison was based on the degree of satisfaction of the user towards both explanations which was measured with the two Likert scale questions 1 and 2. The answers to these questions are summarized in Figure 4. We can clearly notice, in recommendations 1 and 2, the abundance of the “Strongly agree” and “Somewhat agree” answers in “Explanation 1” compared to “Explanation 2.” In fact, in recommendations 1 and 2 respectively, 18 (60%) and 16 (61.5%) users agree that explanation 1 is relevant against only 15 (50%) and 12 (46.1%) that agree the same for explanation 2. However, for the third recommendation, explanation 2 was more relevant than explanation 1 (16 agreeing participants in “Explanation 1” versus 19 in “Explanation 2”). This is probably due to the decreasing relevance of the recommendations in general as we go down in the ranked list of recommendations.

#### 5.4.2 RQ6: Does Our Type of Explanation (Instrumental Segment) Impact the Perceived Effectiveness?

**FIGURE 5:**
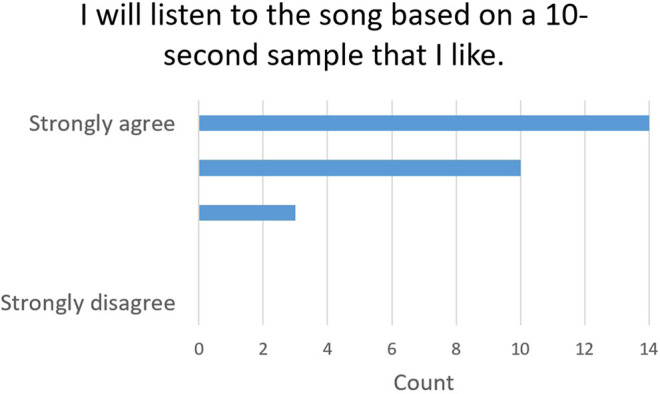
Explainability effectiveness evaluation: Answers to Question 3.

In order to study the effectiveness of our explainability process, we asked the users if they would listen to a song based on a 10-s segment that they like (Question 3). The users almost unanimously agreed (88.9%), among which 51.9% strongly agreed, with no participants disagreeing. This validates the effectiveness of our 10-s segment explainability method. The answers to Question 3 are summarized in Figure 5.

#### 5.4.3 RQ7: Does Our Type of Explanation Increase the Perceived Transparency?

**FIGURE 6:**
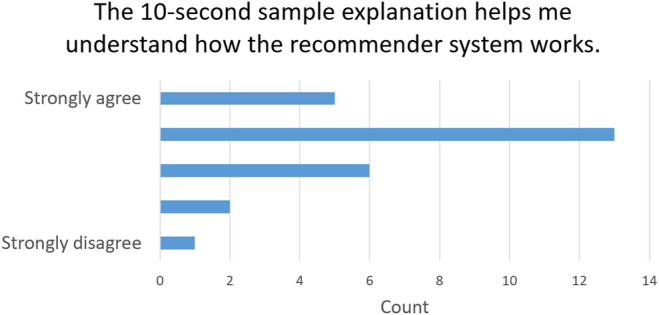
Explainability transparency evaluation: Answers to Question 4.

Finally, to evaluate the impact of our explainability method in terms of transparency, we asked the users whether the 10-s segment explanation helps them understand how the recommender system works (Question 4). 18 (66.7%) users agree that the explanation improves the transparency of system [5 of them (18.5%) strongly agree]. This proves that our explanation helps the user understand how our deep learning model works. The answers to Question 4 are summarized in Figure 6.

## 6 Conclusion

We proposed a hybrid song recommender system (SeER) that combines the user ratings with the songs’ MIDI content to generate both song recommendations and short MIDI segments that serve as *personalized* explanations for each recommended song. In addition to being the only model that can explain its recommendations, SeER stood out among the top performers in terms of recommendation accuracy, compared to baseline recommender systems. The personalized explanation segments’ quality was validated by the fact that they share common properties that capture the user preferences. Moreover, the online survey-based user study validated the approach in terms of relevance, effectiveness and transparency. In addition to its good accuracy and explanation ability, SeER can handle the item cold start problem. In the future, we plan to extend our evaluation and to perform user-based studies.

## Data Availability

The dataset generated and used in this article is made available, along with the source code and some pre-trained models, in the Github repository of the project: https://github.com/KhalilDMK/SeER_Keras.

## Footnotes

1
https://cecm.indiana.edu/361/midi.html

2
http://www.fourmilab.ch/webtools/midicsv/

3
https://drive.google.com/open?id=1E5SZ3I6WNKFTSIodyuzYG9S_Szppz0I9

4
https://github.com/KhalilDMK/SeER_Keras

5
https://paperswithcode.com/sota/collaborative-filtering-on-million-song

6 This user study was performed with a slightly different version of SeER, in which the output layer is a dot product of the user and song latent representations. This was an old version of the model that we updated because of a significant gain in performance that we obtained with the current output layer. Even with this difference in the output layer, the model architecture and the explainability process are the same. Hence, any conclusion drawn from the online experiments with the old version of the model could be extended to the current model. We would also like to mention that we were unable to continue the user study, or replicate it with the updated model, because of the in-person setting of the experiment that was prevented by the COVID-19 pandemic.
